# Increased functional connectivity of the posterior cingulate cortex with the lateral orbitofrontal cortex in depression

**DOI:** 10.1038/s41398-018-0139-1

**Published:** 2018-04-25

**Authors:** Wei Cheng, Edmund T. Rolls, Jiang Qiu, Xiongfei Xie, Dongtao Wei, Chu-Chung Huang, Albert C. Yang, Shih-Jen Tsai, Qi Li, Jie Meng, Ching-Po Lin, Peng Xie, Jianfeng Feng

**Affiliations:** 1 Institute of Science and Technology for Brain-inspired Intelligence, Fudan University, Shanghai, 200433 China; 2 Department of Computer Science, University of Warwick, Coventry, CV4 7AL UK; 3 Oxford Centre for Computational Neuroscience, Oxford, UK; 4 Key Laboratory of Cognition and Personality (SWU), Ministry of Education, Chongqing, China; 5 Department of Psychology, Southwest University, Chongqing, China; 6 Department of Radiology, The First Affiliated Hospital of Chongqing Medical University, Chongqing, 400016 China; 7 Institute of Neuroscience, National Yang-Ming University, Taipei, Taiwan; 8 Department of Psychiatry, Taipei Veterans General Hospital, Taipei, Taiwan; 9 Department of Neurology, The First Affiliated Hospital of Chongqing Medical University, Chongqing, China; 10 Brain Research Center, National Yang-Ming University, Taipei, Taiwan; 11 Institute of Neuroscience, Chongqing Medical University, Chongqing, China; 12 Chongqing Key Laboratory of Neurobiology, Chongqing, China; 13 School of Mathematical Sciences, School of Life Science and the Collaborative Innovation Center for Brain Science, Fudan University, Shanghai, 200433 China

## Abstract

To analyze the functioning of the posterior cingulate cortex (PCC) in depression, we performed the first fully voxel-level resting state functional-connectivity neuroimaging analysis of depression of the PCC, with 336 patients with major depressive disorder and 350 controls. Voxels in the PCC had significantly increased functional connectivity with the lateral orbitofrontal cortex, a region implicated in non-reward and which is thereby implicated in depression. In patients receiving medication, the functional connectivity between the lateral orbitofrontal cortex and PCC was decreased back towards that in the controls. In the 350 controls, it was shown that the PCC has high functional connectivity with the parahippocampal regions which are involved in memory. The findings support the theory that the non-reward system in the lateral orbitofrontal cortex has increased effects on memory systems, which contribute to the rumination about sad memories and events in depression. These new findings provide evidence that a key target to ameliorate depression is the lateral orbitofrontal cortex.

## Introduction

Depression characterized by persistently sad or depressed mood is a major personal burden to sufferers and their families, and is a major economic burden to society^1^. Neuroimaging studies have highlighted a number of large-scale functional networks, such as the default mode network (DMN)^2^, the salience network^3^, the frontoparietal network^4^, and also the interactions between these networks, contributing to the pathophysiology of depression, as recently reviewed^5^. Considerable evidence has shown the important role of the DMN in the pathophysiology of major depression and it appears to be related to rumination and depression severity in MDD^6^.

The posterior cingulate cortex is a key region of the default mode network with strong connectivity in primates with the entorhinal cortex and parahippocampal gyrus, and thus with the hippocampal memory system^7–9^. The posterior cingulate region (including the retrosplenial cortex) is consistently engaged by a range of tasks that examine episodic memory including autobiographical memory, and imagining the future; and also spatial navigation and scene processing^10,11^. It has also been shown that the functional connectivity of the posterior cingulate cortex is related to the rumination in depression^12^. Further we detected some voxels in the posterior cingulate cortex and precuneus with different functional connectivity in depression in a study involving very stringent correction for multiple comparisons for every voxel pair across the whole brain, but did not attempt to separate out the posterior cingulate cortex from the precuneus, nor to analyze how the posterior cingulate cortex may be related to depression^13^.

Given this background, the aim of the current investigation was to focus on the posterior cingulate cortex, and to analyze how voxels in it may have altered functional connectivity in depression; how any different functional connectivities in depression may be related to the symptoms of depression; whether there are differences in effective connectivity in unmedicated patients with major depressive disorder; and the effects of medication on functional connectivity in depression.

In this investigation, we performed the first voxel-level resting state functional-connectivity neuroimaging analysis of depression of voxels in the posterior cingulate cortex (PCC) with all other voxels in the brain in a large sample of 336 patients with depression and 350 matched controls. With this large dataset, we are able to analyze every posterior cingulate voxel for significantly different functional connectivity with every voxel throughout the rest of the brain in depressed people vs controls, in order to advance understanding of the posterior cingulate cortex and depression. In this paper we utilize what we term “hypothesis-based voxel-level functional connectivity analysis” in which we select a brain region of interest, but then calculate for every voxel in that region whether it has functional connectivity with individual voxels in every other brain region. In the present paper, we select the posterior cingulate cortex as the region of interest, given the research on it described above implicating it in depression, and then we show exactly which posterior cingulate voxels have altered functional connectivity in depression with which individual voxels in every other brain area. The advantage over previous seed-wise approaches is that this new method enables identification of which voxels within the posterior cingulate have altered connectivity with other brain regions. Given that the posterior cingulate cortex has 241 voxels, and that there are 47619 3 × 3 × 3 mm^3^ voxels in the automated anatomical atlas (AAL2) brain^14^, the number of voxel pairs in this study was approximately (241 × 47619/2). This methodology is quite different from, and more statistically powerful than, a whole brain voxel-to voxel functional connectivity analysis, which, because there are so many voxels pairs in the whole brain, is rather insensitive, for it carries a huge burden to correct for the multiple comparisons (1,133,760,771 voxel pairs requiring normally *p* < 10^−8^ for any effect to be significant^13^). The AAL2 atlas^14^ was used to provide names for brain areas in which voxels were found, and to define the posterior cingulate cortex region. We note that the AAL2 region identified as PCC is mainly what has been termed ventral posterior cingulate cortex (vPCC)^15^. The PCC is an important site of convergence of the dorsal and ventral processing streams, as it receives from medial and lateral parietal cortical areas, and from temporal cortical areas^8,9,15^. Further, there are direct connections between the parahippocampal gyrus and the PCC, providing a major route via PCC into hippocampal circuitry^9^. The vPCC is activated during self-reflection and self-imagery^16–19^. The PCC also has direct connections from the orbitofrontal cortex^8,9,15^.

The focus here is on the posterior cingulate cortex, because not only is it implicated in depression, as described above, but also it is implicated in emotion and processes fundamental to emotion such as the processing of rewards and non-rewards^20–22^. We relate the discoveries described here to a new theory of depression in which an area that projects to the posterior cingulate cortex, the lateral orbitofrontal cortex, has increased sensitivity of a non-reward attractor in depression; and the medial orbitofrontal cortex reward system is underactive in depression^23,24^.

## Methods

### Participants

There were 336 patients with a diagnosis of major depression, and 350 controls. The patients were from Taiwan (Veteran General Hospital, Taipei) and Xinan (First Affiliated Hospital of Chongqing Medical School in Chongqing,China). All participants were diagnosed according to the Diagnostic and Statistical Manual of Mental Disorder-IV criteria for major depressive disorder. Depression severity and symptomatology were evaluated by the Hamilton Depression Rating Scale (HAMD, 17 items)^25^ and the Beck Depression Inventory (BDI)^26^. In total 211 patients were receiving medication at the time of the neuroimaging. The medication consisted in most cases of selective serotonin reuptake inhibitors (SSRIs) including fluoxetine, paroxetine, sertraline, citalopram, and escitalopram; or serotonin-norepinephrine reuptake inhibitors (SNRIs) such as venflaxine, or a tetracyclic antidepressant such as mirtazepine. Table S2 provides a summary of the demographic information and the psychiatric diagnosis of the participants, and fuller information is provided in Supplementary Material.

### Image acquisition and preprocessing

Data for resting state functional connectivity analysis were collected in 3T MRI scanners in an 8 min period in which the participants were awake in the scanner not performing a task using standard protocols described in Supplementary Material. Data preprocessing was standard, as has been described before^13^, and details are provided in Supplementary Material.

### Hypothesis-based voxel-wise association studies

In the present study, each resting-state fMRI volume included 47,619 voxels, and the posterior cingulate cortex region of interest had 241 voxels in the AAL2 atlas^14^. For each pair of voxels in the cingulate cortex and voxels in all other brain areas, the time series were extracted and their Pearson correlation was calculated for each subject, to provide the measure of functional connectivity, followed by z-transformation. Two-tailed, two-sample *t*-tests were performed on the Fisher’s *z*-transformed correlation coefficients to identify significantly altered functional connectivity links in patients with depression compared to controls within each imaging center. The effects of age, gender ratios, head motion (mean framewise displacements (FD)) and education were regressed out within each dataset in this step by a generalized linear model^27,28^. After obtaining the *t*-test results (*p* value for each functional connectivity) for each center, the Liptak–Stouffer *z* score method^29^ (described in detail in previous studies^13,30,31^) was then used to combine the results from the individual datasets. (With this approach, no dummy covariates for the dataset sites are needed.) Specifically, the *p*-value of each functional connectivity resulting from the two-sample *t*-test in the previous step was converted to its corresponding z score. This was calculated firstly as in equation: $z_k^\prime = {\mathrm{\Phi }}^{ - 1}(1 - p_k)$, where Φ is the standard normal cumulative distribution function and *k* represent the *k* center. Next, a combined *z* score for a functional connectivity was calculated using the Liptak–Stouffer formula: $Z\, = \,{\sum} {w_kz_k^\prime } /{\sum} {w_k^2}$, where $w_k\, = \,\sqrt {sample\,size}$ is the weight of the *k*’th dataset.

Finally, the *Z* is transformed into its corresponding p-value, and a FDR procedure was used to correct for multiple comparisons across the (241 × 47619/2) voxel pairs. The results are presented based on this statistical test with FDR *p* < 0.05, corresponding to a p threshold of 4.4×10^−5^ in the Z-tests.

### Visualization of the differences in functional connectivity (FC) for each voxel

To illustrate in some of the Figures the extent to which voxels in different brain areas had differences of FC between patients and controls, we used a measure for the association (*MA*) between a voxel *i* and the brain disorder. This was defined as: MA=N_α_, where *N*_α_ is the number of links between voxel *i* and every other voxel in the brain that have a *p*-value of less than *α* (in the present study *α*=4.4×10^−5^) in z-tests comparing patients with controls. A larger value of *MA* implies a more significant difference in functional connectivity.

### Code availability

The code is available upon request to one of the two first authors.

### Clinical correlates

We also investigated whether the differences in functional connectivity (FC) between patients and controls were correlated with clinical variables (the Hamilton depression rating Scale (HAMD)^25^, Beck Depression Inventory (BDI)^26^, and illness duration^32,33^). We used the Liptak–Stouffer z-score method^29^ to combine the data from the different neuroimaging sites for this analysis, for this provides a principled way to take into consideration possible differences in these measures between sites. Specifically, the functional connectivity of the voxels with significant differences of functional connectivity (after FDR correction at *p* < 0.05, and within the voxel clusters shown in Table 1) was measured for each of the AAL2 regions within which the voxels were located. In this way, 17 regions of interest (ROI) were identified. Then we calculated the partial correlation between the ROI-wise FCs and the clinical scores, with head motion, education, sex, age and medication as covariates so that they did not contribute to the correlation between the FCs and the clinical scores, in each individual center. Then we used the Liptak-Stouffer z-score method to combine the results from the individual datasets^29^.

**Table 1:** Numbers of voxels in different AAL2 areas with significantly different functional connectivity with the posterior cingulate cortex (PCC) in patients with depression

Areas	Sum z value	# Voxels	MNI coordinates (Peak, *X* *Y* *Z*)		
OFCant_R, OFCpost_R	197.8	12	39	36	−15
Frontal_Inf_Orb_2_R, OFClat_R	205.7	30	36	36	−12
Cingulate_Ant_L, Cingulate_Ant_R	456.5	170	−3	36	3
Cuneus_L, Cuneus_R, Precuneus_L	52.0	30	−9	−60	21
Temporal_Pole_Mid_L	112.7	23	−33	18	−39
Occipital_Inf_L, Temporal_Inf_L	4.3	23	−51	−60	−12
Fusiform_L, Fusiform_R	50.8	78	−33	−42	−18
Frontal_Mid_2_R, Frontal_Inf_Tri_R	84.4	24	54	48	−3
Frontal_Sup_Medial_L, Frontal_Sup_Medial_R	39.5	40	−3	60	9
Cingulate_Post_L, Cingulate_Post_R	926.5	203	−9	−45	30
Frontal_Inf_Oper_R, Frontal_Inf_Tri_R	722.5	120	36	12	27
Frontal_Sup_2_L, Frontal_Mid_2_L	52.0	26	−30	21	36
Frontal_Mid_2_R	34.8	19	42	15	54

For PCC, the table shows the number of PCC voxels that have different functional connectivity with the whole brain (FDR corrected, *p* < 0.05). The other entries in the table show the numbers of voxels in each of the specified brain regions with different functional connectivity with PCC voxels (FDR corrected, *p* < 0.05). The z value is the sum across voxels and FC links of the absolute value of the z score for a significant link between a pair of voxels. Here, we only show clusters with more than 10 voxels

## Results

### A voxel-level Association Study (vAS) of posterior cingulate gyrus voxels with different functional connectivity in depressed patients

**Fig. 1:**
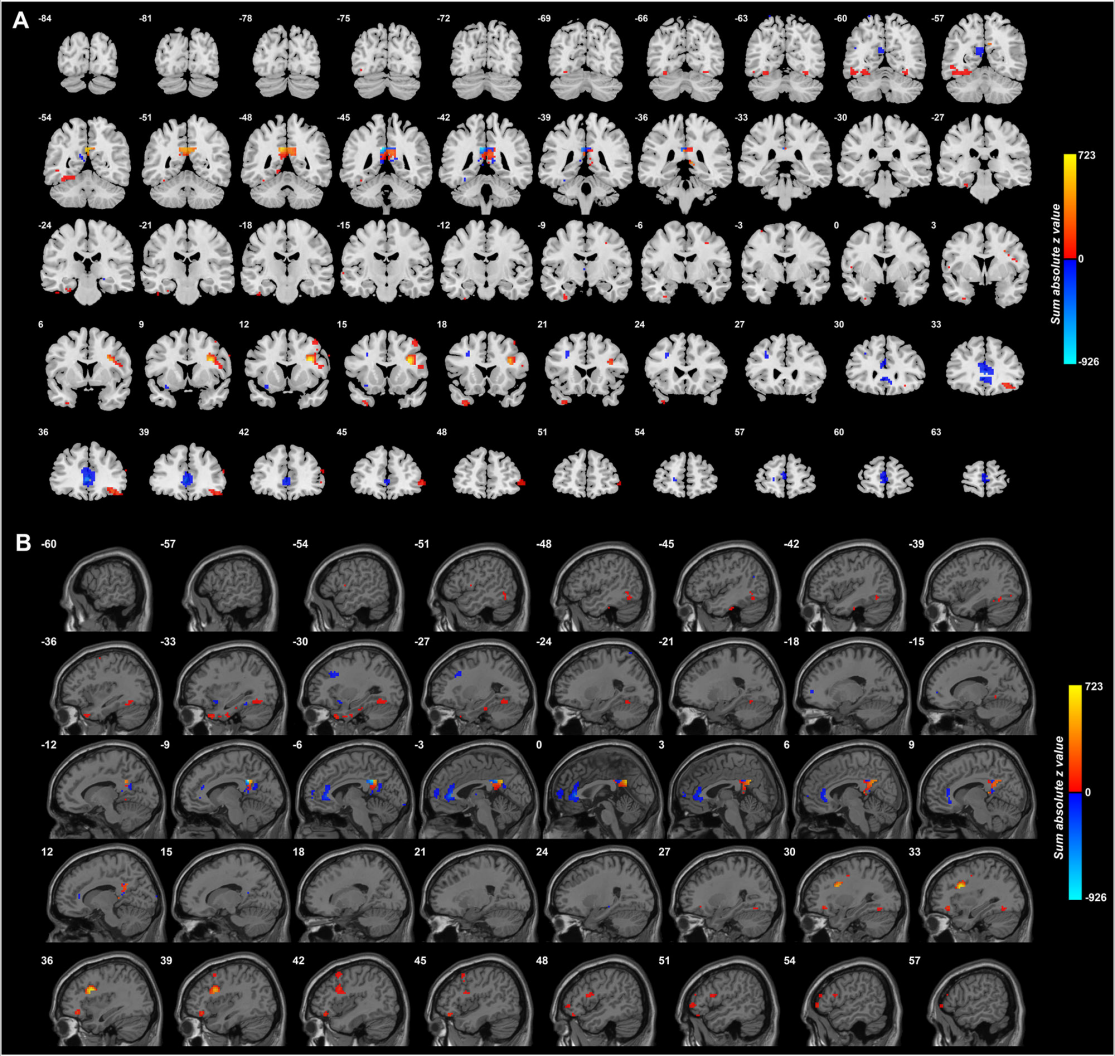
Anatomical location of voxels with significantly different functional connectivity with the posterior cingulate cortex in depression obtained from the voxel-based Association Study (vAS). Data from the whole group of patients, i.e., medicated and unmedicated, were included for this figure. z values are shown for each voxel, showing the mean difference of functional connectivities for patients with depression-controls. Red thus indicates an increase in functional connectivity in depression, and blue a decrease. (The z value shown for each voxel is the sum of the absolute values of the z for each significantly different link to a voxel, with a red color if the sum of the signed values is positive. This enables the effects of different numbers of different links to a voxel to be illustrated. Links are considered only if they are significantly different with FDR correction with *p* < 0.05.) The right of the brain is on the right of each slice. The *Y* and *X* values are in MNI coordinates. **a** Coronal slices. **b** Parasagittal slices

As shown in Fig. 1 many posterior cingulate cortex voxels (*Y* = −42 to *Y* = −54) had overall increased functional connectivity with voxels in other brain areas in depression. The voxels in other brain areas with increased functional connectivity with the PCC include the lateral orbitofrontal cortex (*Y* = 48 to *Y* = 33); temporal pole (*Y* = 24 to *Y* = 15); inferior frontal gyrus (*Y* = 24 to *Y* = 6); parahippocampal gyrus (*Y* = −9 to *Y* = −27); and the fusiform gyrus (*Y* = −54 to *Y* = −63). There was decreased functional connectivity in depression to some voxels in the anterior cingulate cortex. The whole group of patients was included for this figure.

To investigate the brain areas between which there was different functional connectivity in depression, and whether it was increased or decreased, the functional connectivity (FC) of the posterior cingulate cortex voxels with significant differences of FC (after FDR correction at *p* < 0.05) was measured for each of the AAL2 regions within which the voxels were located. (A list of abbreviations of the AAL2 areas^14^ is provided in Table S1.) The functional connectivity differences are shown in Table 1, which shows the MNI coordinates of the different groups of voxels found in different brain areas that had significantly different FC in depression, the numbers of such voxels, and a significance score. The brain areas include the lateral orbitofrontal cortex (AAL2 areas OFClat and Frontal_Inf_Orb_2, with mainly increases of FC, as shown in Fig. 1); the anterior cingulate cortex (decreased FC); the fusiform gyrus (mainly decreased FC); the temporal cortex areas (Temp_Inf and Temp_Pole); the inferior frontal gyrus (Frontal_Inf_Oper, Frontal_Inf_Tri); the middle and superior frontal gyri; occipital areas; the precuneus and cuneus; and middle orbitofrontal cortex (OFC_ant, OFC_post, decreased). The parts of the inferior frontal gyrus are shown in Fig. 1, and the coordinates shown in Table 1 are close to those of the inferior frontal gyrus region with connections with the motor laryngeal area^34^.

### Comparison of the functional connectivity in healthy controls and in patients with depression

**Fig. 2:**
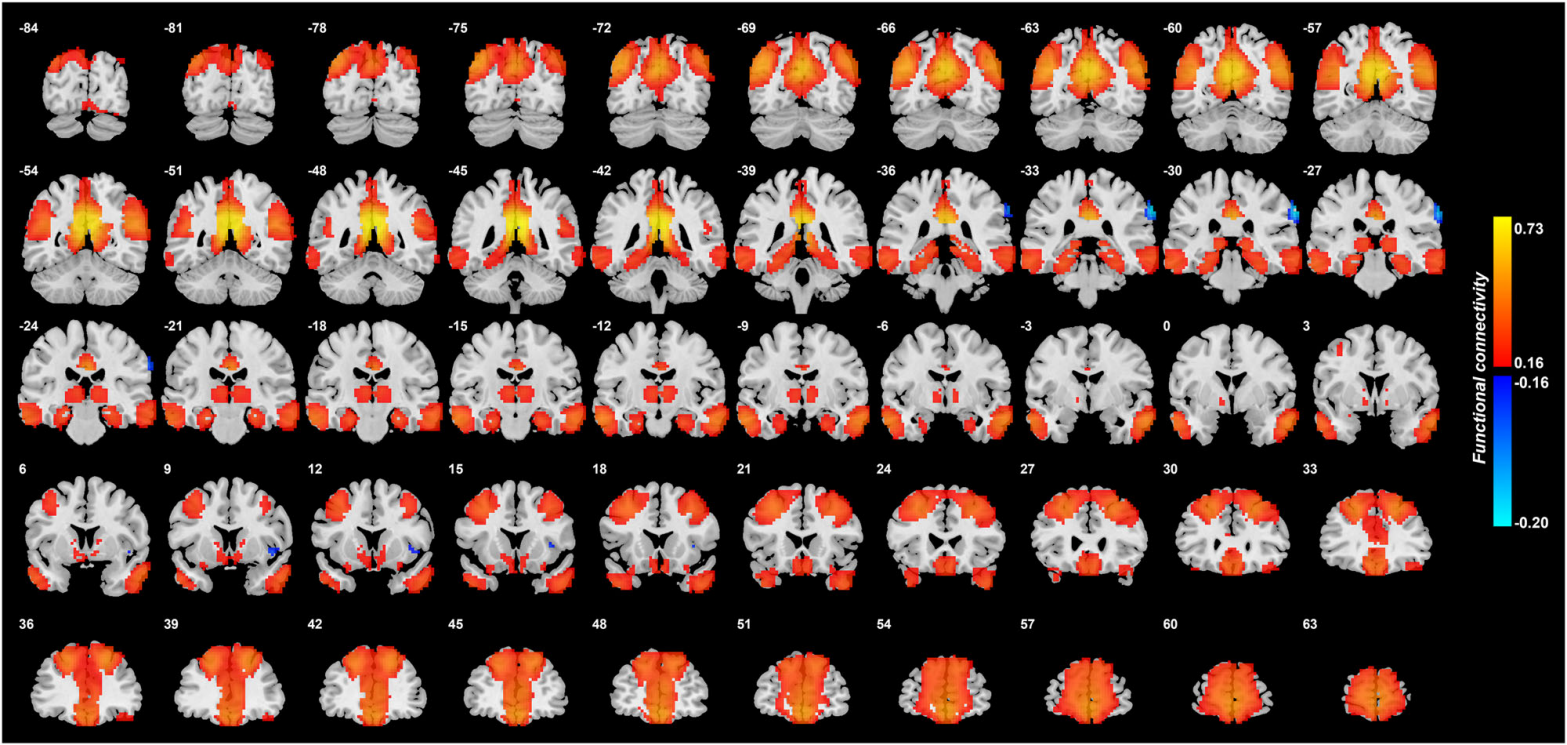
Functional connectivity of the posterior cingulate cortex in 350 healthy controls. *r* values are shown, thresholded at *r* = ± 0.16

Figure 2 shows the functional connectivity between the posterior cingulate cortex and other brain areas in healthy controls. This shows that in controls the PCC has moderately high FC with superior and middle frontal gyri, and a part of the inferior frontal gyrus; the lateral orbitofrontal cortex; the anterior cingulate cortex down into the gyrus rectus; the temporal pole; the hippocampus and parahippocampal areas; the fusiform gyri; and the precuneus and parts of the lateral parietal cortex. Additional information on some of these functional connectivities is shown in Table S3 in the column labeled “FC of controls”.

Table S3 shows a comparison between the functional connectivity in the healthy controls and in all the patients with major depressive disorder (i.e., including medicated and unmedicated patients), for the areas with significant differences. The functional connectivity value shown is the mean r (correlation) value between the significant voxels in the posterior cingulate cortex and all significant voxels in each of the AAL2 areas indicated. For comparison, the mean r value for the FC of the PCC with all other brain areas in the healthy controls is 0.129. Table S3 shows that in healthy controls the PCC has moderately high FC with superior and middle frontal gyri; the lateral orbitofrontal cortex; the anterior cingulate cortex; the fusiform gyri; the precuneus, the temporal pole, and the inferior frontal gyrus regions noted above. Table S3 also shows the mean values in the patients with depression (i.e., including both the medicated and unmedicated patients), and the t and p values of the differences from the healthy controls. Table S3 makes it clear that on average there was in depression (in the whole group of all patients) increased FC (a positive *t* value) of the PCC with the lateral orbitofrontal cortex (AAL2 areas OFClat and Frontal_Inf_Orb_2); with the inferior frontal gyrus (Frontal_Inf_Oper and Frontal_Inf_Tri); and with the temporal pole. Table S3 also makes it clear that on average there was in depression decreased FC (a negative *t* value) of the PCC with the anterior cingulate cortex; the precuneus; and parts of the superior frontal gyrus when the whole depression group including medicated patients is considered.

### Functional connectivity of unmedicated patients with depression

The analyzes shown in Fig. 1 and Table 1 included 125 patients without medication, and 211 with antidepressant medication. Given that all patients from Taiwan were medicated and the sample size was much smaller, the analysis on the effect of medication was performed only with the Xinan dataset. The patients with medication tended to be longer term patients that those without medication, who were in many cases first-episode patients. When we refer to medicated vs unmedicated in what follows, the longevity of the depression may be a factor. Nevertheless, important comparisons can be made as follows.

**Fig. 3:**
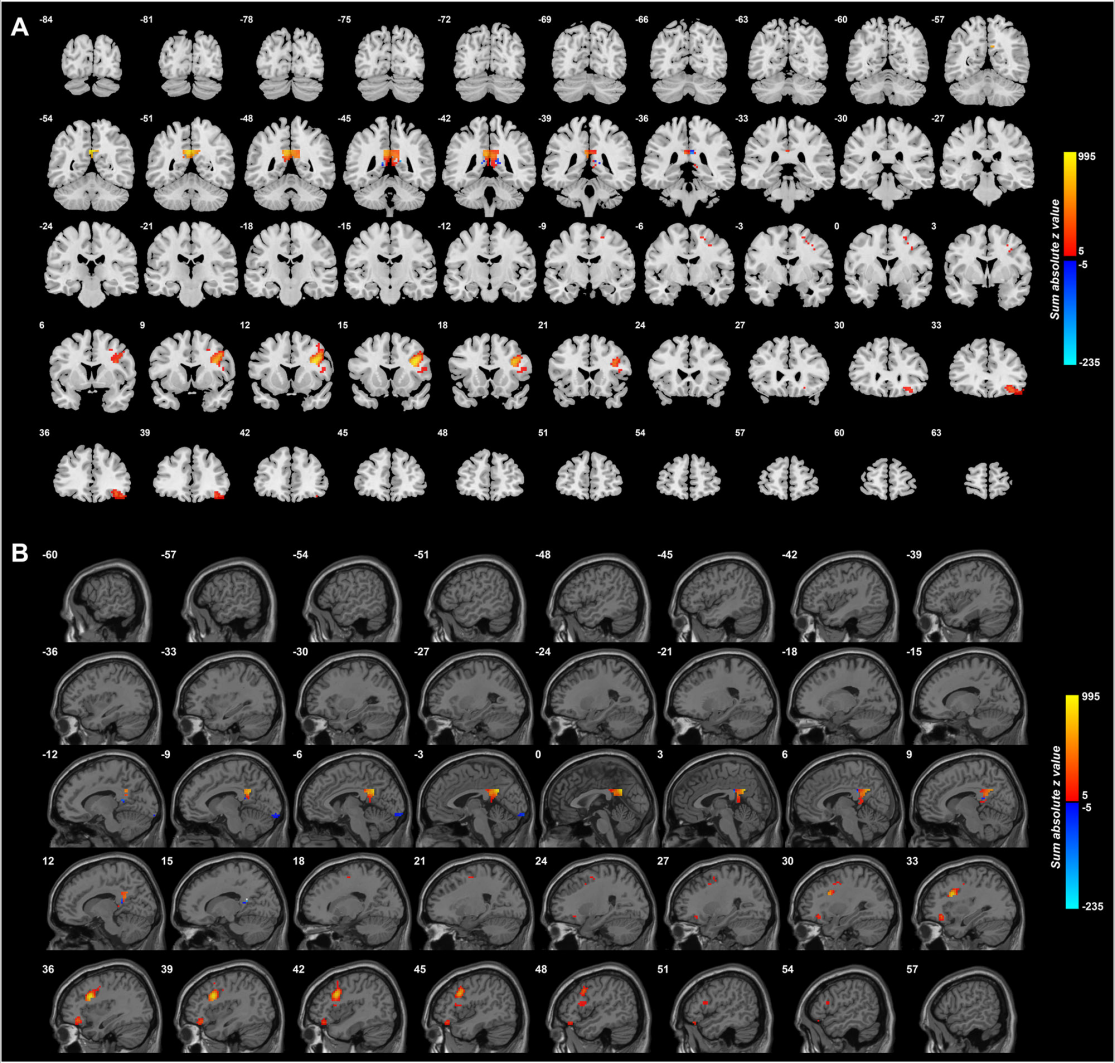
Anatomical location of voxels with significantly different functional connectivity with the posterior cingulate cortex in depression in 125 unmedicated patients vs 254 controls obtained from the voxel-based Association Study (vAS). *z* values are shown for each voxel, showing the mean difference of functional connectivities for patients with unmedicated depression-controls. Red thus indicates an increase in functional connectivity in depression, and blue a decrease. (The *z* value shown for each voxel is the sum of the absolute values of the *z* for each significantly different link to a voxel, with a red color if the sum of the signed values is positive. This enables the effects of different numbers of different links to a voxel to be illustrated. Links are considered only if they are significantly different with *p* < 0.0001.) The *X* values are in MNI coordinates. This shows that the main differences are an increase in functional connectivity between the PCC and the lateral orbitofrontal cortex in depression that is unmedicated

Figure 3 shows the difference in the functional connectivities of the 125 unmedicated patients from the 254 controls. This shows that the main differences are an increase in functional connectivity between the PCC and the lateral orbitofrontal cortex, and between the PCC and inferior frontal gyrus, in unmedicated patients with depression. The lateral orbitofrontal cortex regions is on the right between *X* = 51 and *X* = 27. The inferior frontal gyrus region is shown between *X* = 45 and *X* = 43.

### Effect of medication on functional connectivity involving the posterior cingulate cortex

**Fig. 4:**
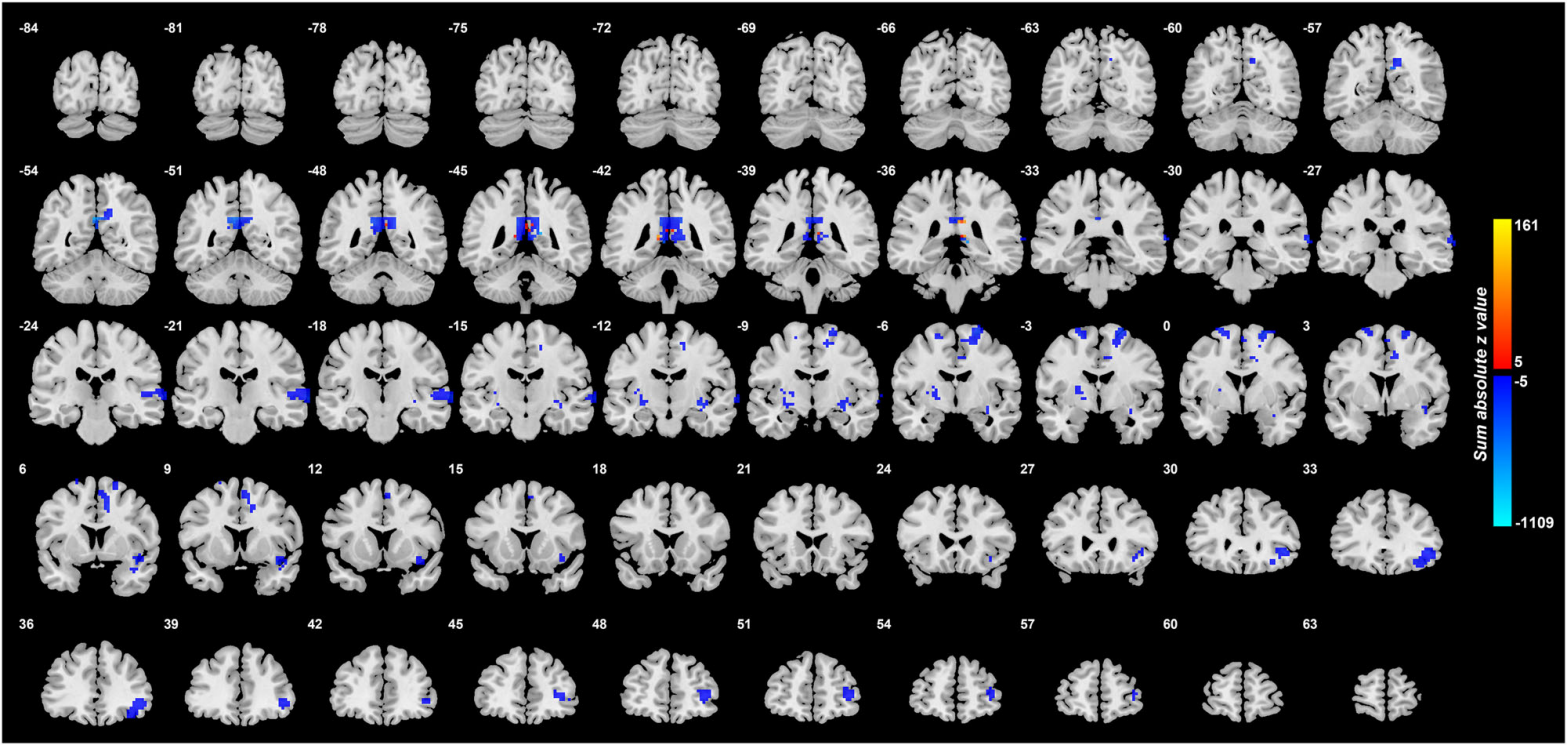
Voxels in the brain with different functional connectivity in the comparison medicated–non-medicated patients with depression. *z* values are shown for each voxel, showing the mean difference of functional connectivities for patients with medicated depression–non-medicated depression. Blue thus indicates a decrease in functional connectivity associated with the medication. (The *z* value shown for each voxel is the sum of the absolute values of the *z* for each significantly different link to a voxel, with a red color if the sum of the signed values is positive. This enables the effects of different numbers of different links to a voxel to be illustrated. Links are considered only if they are significantly different with *p* < 0.001, cluster size > 20 voxels). The right of the brain is on the right of each slice. The *Y* values are in MNI coordinates

To investigate where the medication may influence the functional connectivity, we show in Fig. 4 the voxels with different functional connectivity between the 125 unmedicated patients and the 157 medicated patients. Blue in this diagram indicates a reduction in FC produced by the medication. The medication is associated with a reduction of FC between the lateral orbitofrontal cortex and the PCC. Thus for the lateral orbitofrontal cortex links with increased FC with the PCC in unmedicated depression, the depression reduces those functional connectivities. An implication is that one way in which antidepressants work is by reducing the FC between the lateral orbitofrontal cortex and the PCC.

In addition, the medication reduced the FC of the PCC with the ventral insula (an area implicated in autonomic function); with the middle frontal gyrus (an area implicated in working memory); and with the temporal lobe cortex (Fig. 4).

### Clinical status correlates of the increased posterior cingulate gyrus functional connectivities in depression

To obtain further evidence on whether the functional connectivities that were significantly different in depression, we performed correlation analyzes between the clinical measures and the AAL2 region based functional connectivities. It was found that illness duration was negatively correlated with the decrease in functional connectivity between the PCC and the anterior cingulate cortex (*r* = 0.12, *p* = 0.03). These correlations provide additional evidence closely relating the differences in functional connectivity described here to the depression.

## Discussion

The main findings were as follows. Voxels in the PCC had significantly increased functional connectivity with the lateral orbitofrontal cortex, a region implicated in non-reward and which is thereby implicated in depression. PCC voxels also had increased functional connectivity with the inferior frontal gyrus in depression. In patients receiving medication, the functional connectivity between the lateral orbitofrontal cortex and PCC was increased back towards that in the controls. In the 350 controls, it was shown that the PCC has high functional connectivity with the parahippocampal regions which are involved in memory.

The posterior cingulate cortex (area 23)^35–37^ is a key region related to memory^7^. (It is noted that the adjoining retrosplenial cortex (areas 29 and 30) is included in AAL2 area the precuneus.) It provides connections to and receives connections from the hippocampal system, connecting especially with the parahippocampal gyrus areas TF and TH, and with the subiculum^7,35,36^, with the functional connectivity shown in Fig. 2. The PCC can be conceptualized as providing access to the hippocampus for spatial and related information from the parietal cortex (given the rich connections between the PCC and parietal cortex^8,35,36^ evident in Fig. 2), and with object information from the temporal lobe connecting to and from the hippocampus via the perirhinal cortex^38^. This provides a basis for the hippocampus to associate together object and spatial information in the single network in the CA3 region of the hippocampus, to form an episodic memory with object and spatial components^39^. However, reward-related/emotional information may also be part of an episodic memory, and connections from the orbitofrontal cortex to the hippocampal system via the posterior cingulate cortex, as well as by the perirhinal and entorhinal cortex pathway are the two main routes^22,38,40,41^. Interestingly, the relatively strong functional connectivity between the PCC and the lateral orbitofrontal cortex described here and illustrated in Fig. 2, indicates that reward/punishment-related information also enters this part of the system, and consistently, the parahippocampal region is activated by negative/sad mood induction^42,43^. Indeed, although the PCC can be activated by happy and neutral stimuli in healthy individuals^44,45^, one of the points made here is that the inputs to the PCC may be biased towards unhappy or sad events because of the increased functional connectivity with the lateral orbitofrontal cortex in depression revealed here.

Although a number of areas shown in Fig. 1 had altered functional connectivity in depression with the PCC, it was found that it was primarily the link between the PCC and the lateral orbitofrontal cortex that stood out in unmedicated patients. It was of interest that the medication reduced the FC between the PCC and the lateral orbitofrontal cortex. The other link that stood out in the unmedicated patients was the increased functional connectivity between the PCC and the inferior frontal gyrus region shown in Fig. 3 (and also in Fig. 1 and Table 1 for the whole group of patients) which is probably the inferior frontal gyrus region with connections with the motor laryngeal area^34^. It is suggested that this is related to the increased rumination in depression which may produce subliminal speech-related effects.

To test some of the implications of the findings described here, it would be of interest to perform a functional neuroimaging study in which activations in the PCC and the lateral orbitofrontal cortex to sad vs happy memories are measured. The prediction is that in the PCC and the lateral orbitofrontal cortex the activations would be higher in depressed people for sad vs happy memories.

In a previous study, it was found that some voxels in the lateral orbitofrontal cortex had increased functional connectivity with the precuneus and posterior cingulate cortex, which are nearby and connected brain regions^13^. In the present study we were able to separate out the involvement of the posterior cingulate cortex as follows. An implication is that the increased functional connectivity of the lateral orbitofrontal cortex, a region implicated in non-reward and punishment and therefore related to depression^23,41^, with these posterior regions is to the PCC, a brain region involved in memory. The clear implication is that the lateral orbitofrontal cortex—PCC circuit is related to the enhanced bias towards negative memories and rumination in depression. Indeed, this is complemented by reduced functional connectivity between the medial orbitofrontal cortex, a region implicated in reward, and the parahippocampal gyrus, other main input/output region for hippocampal circuitry^13^. The implication is that there is reduced processing of sad memories related to the medial orbitofrontal cortex—parahippocampal gyrus circuit^13^, and increased processing of sad memories related to the lateral orbitofrontal cortex—PCC circuit in depression, as described here. We propose that these two complementary processes are related to the rumination of negatively valenced thoughts in depression.

The findings described here are also of conceptual interest in relation to brain systems involved in memory and emotion^22,40^. One route for information related to reward and punishment, and therefore to emotion, to gain access to memory systems is via the orbitofrontal cortex projections to the perirhinal cortex and thus to the entorhinal cortex and hippocampus^38,46,47^. Here we show functional connectivity between the lateral orbitofrontal cortex and posterior cingulate cortex, which via its connections to the parahippocampal gyrus and entorhinal cortex, provides a second route for reward and punishment-related information involved in emotion, especially from the lateral orbitofrontal cortex, to reach the hippocampal memory system. Moreover, we show here that this lateral orbitofrontal cortex to posterior cingulate cortex system has enhanced functional connectivity in depression, which may contribute to the sad ruminating thoughts in depression.

The importance of the present study is that by focusing on the posterior cingulate cortex, and using very large neuroimaging datasets of patients with depression and controls, we were able to characterize the altered functional connectivity at the voxel level in depression of the posterior cingulate cortex with other brain regions. A strength of this investigation is that we analyzed functional connectivity at the level of voxel to voxel functional connectivity. This was made possible by the uniquely large sample size, which enabled the conclusions described above to be reached. The findings support the theory that the non-reward system in the lateral orbitofrontal cortex has increased effects on memory systems, which contribute to the rumination about sad memories and events in depression^23,24,41,48^. These new findings provide further evidence that a key target to ameliorate depression is the lateral orbitofrontal cortex^23,41,48,49^.

## Electronic supplementary material

Supplemental material
