# Supplementary material for: Increased functional connectivity of the posterior cingulate cortex with the lateral orbitofrontal cortex in depression
Source: Transl Psychiatry. 2018 Apr 25;8:90. doi: 10.1038/s41398-018-0139-1 (PMC5915597; doi:10.1038/s41398-018-0139-1)
Supplement: Supplementary file 1 — Supplemental material [file 41398_2018_139_MOESM1_ESM.docx]

**Increased functional connectivity of the** **posterior cingulate cortex with the lateral orbitofrontal cortex in depression**

Wei Cheng^1,#^; Edmund T. Rolls^2,3,#^; Jiang Qiu^4,5,#^; Xiongfei Xie^13,#^; Dongtao Wei^5^; Chu-Chung Huang^7^; Albert C. Yang^11^; Shih-Jen Tsai^11^; Qi Li^10^; Jie Meng^5^; Ching-Po Lin^1,6,7,*^; Peng Xie^8,9,10,*^; Jianfeng Feng^1, 2, 12,*^

1. Institute of Science and Technology for Brain-inspired Intelligence, Fudan University, Shanghai, 200433, China

2. Department of Computer Science, University of Warwick, Coventry CV4 7AL, UK

3. Oxford Centre for Computational Neuroscience, Oxford, UK

4. Key Laboratory of Cognition and Personality (SWU), Ministry of Education, Chongqing, China

5. Department of Psychology, Southwest University, Chongqing, China

6. Brain Research Center, National Yang-Ming University, Taipei, Taiwan

7. Institute of Neuroscience, National Yang-Ming University, Taipei, Taiwan

8. Institute of Neuroscience, Chongqing Medical University, Chongqing, China

9. Chongqing Key Laboratory of Neurobiology, Chongqing, China

10. Department of Neurology, The First Affiliated Hospital of Chongqing Medical University, Chongqing, China

11. Department of Psychiatry, Taipei Veterans General Hospital, Taipei, Taiwan

12. School of Mathematical Sciences, School of Life Science and the Collaborative Innovation Center for Brain Science, Fudan University, Shanghai, 200433, PR China

13. Department of Radiology,the First Affiliated Hospital of Chongqing Medical University, Chongqing 400016,China

^#^ These authors contributed equally to this work.

**Participants**

There were 336 patients with a diagnosis of major depression, and 350 controls. The patients were from Xinan (First Affiliated Hospital of Chongqing Medical School in Chongqing, China), and Taiwan (Veteran General Hospital, Taipei). All participants were diagnosed according to the Diagnostic and Statistical Manual of Mental Disorder-IV criteria for major depressive disorder. Depression severity and symptomatology were evaluated by the Hamilton Depression Rating Scale (HAMD, 17 items) ^1^ and the Beck Depression Inventory (BDI) ^2^. Table S0 provides a summary of the demographic information and the psychiatric diagnosis (showing how they were diagnosed) of the participants. The data collection was approved by the local ethical review committees, was in accordance with the Code of Ethics of the World Medical Association (Declaration of Helsinki), and informed consent was obtained. This is a subset of patients from our previous functional connectivity investigation ^3^, but the analysis used here is completely different and novel in its application to depression, for it assesses effective or directed connectivity, and goes beyond correlations. With respect to age and sex, Table S2 shows that there were no significant differences in the age and sex of the depressed groups and the controls. Further, the effects of age and sex were regressed out in all analyses. 125 of the patients were not receiving medication at the time of the neuroimaging. Further details follow.

*Xinan:* Patients with MDD were recruited from the outpatient department of the First Affiliated Hospital of Chongqing Medical School in Chongqing, China. All were diagnosed according to the Structured Clinical Interview for DSM-IV, by independent assessments of two psychiatrists. They were also assessed for disease severity using the Hamilton Depression Rating Scale (HAMD) ^1^ and Beck Depression Inventory (BDI), illness duration and the medication status of the patients. Before the investigation, we excluded individuals who were not suitable for MRI scanning by interview and by the self-reported checklist. The MRI related exclusion criteria include claustrophobia, metallic implants, Meniere’s Syndrome and a history of fainting within the previous half year. Exclusion criteria for both groups were as follows: current psychiatric disorders (except for MDD) and neurological disorders; substance abuse; and stroke or serious encephalopathy. Of note, all of the subjects in the control group did not meet DSM-IV criteria for any psychiatric disorders and did not use any drugs that could affect brain function. This study was approved by the Research Ethics Committee of the Brain Imaging Center of Southwest University and First Affiliated Hospital of Chongqing Medical School. Informed written consent was obtained from each subject. This study was conducted in accordance with the Helsinki Declaration as revised in 1989.

*Taiwan:* Patients were recruited from the Veteran General Hospital in Taipei, Taiwan. All participants were diagnosed according to the Diagnostic and Statistical Manual of Mental Disorder-IV criteria for depression, and each participant's history of medical disease, psychiatric illness, and medication use was evaluated by interview and medical charts carefully. Experiments were conducted in accordance with the Declaration of Helsinki and approved by the Institutional Review Board of Taipei Veterans General Hospital. Written informed consent was obtained from all participants after ensuring adequate understanding of the study. Any participants with the following conditions were excluded: (1) a comorbid substance-related disorder, (2) presence of neurobiological disorders, such as dementia, head injury, stroke, or Parkinson’s disease; (3) presence of hypertension, diabetes, hyperlipidemia or coronary heart disease; (4) severe medical illness, such as malignancy, heart failure, or renal failure; (4) presence of ferromagnetic foreign bodies or implants that were anywhere in the body. Depression severity was evaluated by the psychiatrist-assessed Hamilton Depression Rating Scale (HAMD, 17 items) ^1^.

**Image Acquisition**

*Xinan:* All images were acquired on a 3.0-T Siemens Trio MRI scanner using a 16-channel whole-brain coil (Siemens Medical, Erlangen, Germany). High- resolution T1-weighted 3D images were acquired using a magnetization-prepared rapid gradient echo (MPRAGE) sequence (echo time (TE) = 2.52 ms; repetition time (TR) = 1900 ms; inversion time (TI) = 900 ms; flip angle = 9 degrees; slices = 176; thickness = 1.0 mm; resolution matrix = 256×256; voxel size = 1×1×1 mm3). For each participant, 242 functional images were acquired with a gradient echo type Echo Planar Imaging (EPI) sequence (echo time (TE) = 30 ms; repetition time (TR) = 2000 ms; flip angle = 90 degrees; slices = 32; slice thickness = 3.0 mm; slice gap = 1 mm; resolution matrix = 64×64; voxel size 3.4×3.4×3〖mm〗^3). During image acquisition, participants were instructed to keep their eyes closed while keeping their head as still as possible without falling asleep. All participants stayed awake during the MRI imaging as confirmed by the participants after the session.

*Taiwan:* fMRI scanning was performed at National Yang-Ming University in Taiwan using a 3.0-T Siemens MRI Scanner (Siemens Magnetom Tim Trio, Erlangen, Germany) with a 12-channel head coil. During the experiments, the participants were instructed to relax with their eyes closed, without falling asleep. After the resting state experiment, participants were asked whether they fell asleep during the resting state scan session, and participants were rescanned if they had fallen asleep during the resting state scan. T2*-weighted images with BOLD contrast were measured using a gradient echo- planar imaging (EPI) sequence (repetition time, TR: 2,500 ms, echo time, TE: 27 ms, field of view, FoV: 220 mm, flip angle: 77 degree, matrix size: 64 x 64, and voxel size: 3.44×3.44×3.40 mm). For each run, 200 EPI volume images were acquired in the anterior and posterior commissure (AC–PC) plane. High-resolution structural T1 images were acquired with three-dimensional (3D) magnetization-prepared rapid gradient- echo sequence (3D-MPRAGE; TR: 2,530 ms, TE: 3.5 ms, TI: 1,100 ms, FoV: 256 mm, and flip angle: 7 degree, 192 sagittal slices, voxel size = 1.0 mm x 1.0 mm 1.0 mm, no gap). For each participant, the whole fMRI scanning lasted about 16 min (T1: 8min, Resting: 8min).

**Data Preprocessing**

Data for resting state functional connectivity analysis were collected in 3T MRI scanners in an 8 min period in which the participants were awake in the scanner not performing a task using standard protocols described below.

Data preprocessing was performed using DPARSF ^4^ (http:// restfmri.net) which is a toolbox based on the SPM8 software package. The first 10 EPI scans were discarded to suppress equilibration effects. The remaining scans of each subject underwent slice timing correction by sinc interpolating volume slices, motion correction for volume to volume displacement, spatial normalization to standard Montreal Neurological Institute (MNI) space using affine transformation and nonlinear deformation with a voxel size of $3\times3\times3{mm}^{3}$, followed by spatial smoothing (8 mm Full Width Half Maximum FWHM). To remove the sources of spurious correlations present in resting-state BOLD data, all fMRI time-series underwent band-pass temporal filtering (0.01-0.1 Hz), nuisance signal removal from the ventricles, and deep white matter, and regressing out any effects of head motion using the Friston et al 24 head motion parameters procedure ^5^. Finally, we implemented additional careful volume censoring (“scrubbing”) movement correction as reported by Power et al. ^6^ to ensure that head-motion artifacts are not driving observed effects. The mean framewise displacement (FD) was computed with FD threshold for displacement being 0.5. In addition to the frame corresponding to the displaced time point, 1 preceding and 2 succeeding time points were also deleted to reduce the spill-over effect of head movements. Subjects with >10% displaced frames flagged were completely excluded from the analysis as it is likely that such high-level of movement would have had an influence on several volumes. Global signals were not regressed out, for reasons described elsewhere ^3^. Considering the potential effect of gender ^7^, age ^8^ and head motion ^6, 9^ on functional connectivity, any effects of gender ratio, years of education, age and head motion between the patient and control groups were regressed out in all analyses. There were no differences in the gender ratios, age and mean FD (p>0.05 in all cases), though the number of years of education was lower in the patients than controls. However, none of the functional connectivity link differences found between patients and controls was correlated significantly (FDR p<0.05) with the number of years of education. We also note that the Taiwanese sample included patients with depression in remission while under antidepressant treatment, and thus their scores on the Hamilton Depression Rating Scale (HAMD) assessment were in the low range.

**Table S1.** The anatomical regions defined in each hemisphere and their label in the automated anatomical labelling atlas AAL2 ^10^. Column 4 provides a set of possible abbreviations for the anatomical descriptions.

| NO. | ANATOMICAL DESCRIPTION | LABEL  aal2.nii.gz | POSSIBLE  ABBREVIATION |
| --- | --- | --- | --- |
| 1,2 | Precentral gyrus | Precentral | PreCG |
| 3, 4 | Superior frontal gyrus, dorsolateral | Frontal_Sup | SFG |
| 5, 6 | Middle frontal gyrus | Frontal_Mid | MFG |
| 7, 8 | Inferior frontal gyrus, opercular part | Frontal_Inf_Oper | IFGoperc |
| 9, 10 | Inferior frontal gyrus, triangular part | Frontal_Inf_Tri | IFGtriang |
| 11, 12 | IFG pars orbitalis, | Frontal_Inf_Orb | IFGorb |
| 13, 14 | Rolandic operculum | Rolandic_Oper | ROL |
| 15, 16 | Supplementary motor area | Supp_Motor_Area | SMA |
| 17, 18 | Olfactory cortex | Olfactory | OLF |
| 19, 20 | Superior frontal gyrus, medial | Frontal_Sup_Med | SFGmedial |
| 21, 22 | Superior frontal gyrus, medial orbital | Frontal_Med_Orb | PFCventmed |
| 23, 24 | Gyrus rectus | Rectus | REC |
| 25, 26 | Medial orbital gyrus | OFCmed | OFCmed |
| 27, 28 | Anterior orbital gyrus | OFCant | OFCant |
| 29, 30 | Posterior orbital gyrus | OFCpost | OFCpost |
| 31, 32 | Lateral orbital gyrus | OFClat | OFClat |
| 33, 34 | Insula | Insula | INS |
| 35, 36 | Anterior cingulate & paracingulate gyri | Cingulate_Ant | ACC |
| 37, 38 | Middle cingulate & paracingulate gyri | Cingulate_Mid | MCC |
| 39, 40 | Posterior cingulate gyrus | Cingulate_Post | PCC |
| 41, 42 | Hippocampus | Hippocampus | HIP |
| 43, 44 | Parahippocampal gyrus | ParaHippocampal | PHG |
| 45, 46 | Amygdala | Amygdala | AMYG |
| 47, 48 | Calcarine fissure and surrounding cortex | Calcarine | CAL |
| 49, 50 | Cuneus | Cuneus | CUN |
| 51, 52 | Lingual gyrus | Lingual | LING |
| 53, 54 | Superior occipital gyrus | Occipital_Sup | SOG |
| 55, 56 | Middle occipital gyrus | Occipital_Mid | MOG |
| 57, 58 | Inferior occipital gyrus | Occipital_Inf | IOG |
| 59, 60 | Fusiform gyrus | Fusiform | FFG |
| 61, 62 | Postcentral gyrus | Postcentral | PoCG |
| 63, 64 | Superior parietal gyrus | Parietal_Sup | SPG |
| 65, 66 | Inferior parietal gyrus, excluding supramarginal and angular gyri | Parietal_Inf | IPG |
| 67, 68 | SupraMarginal gyrus | SupraMarginal | SMG |
| 69, 70 | Angular gyrus | Angular | ANG |
| 71, 72 | Precuneus | Precuneus | PCUN |
| 73, 74 | Paracentral lobule | Paracentral_Lobule | PCL |
| 75, 76 | Caudate nucleus | Caudate | CAU |
| 77, 78 | Lenticular nucleus, Putamen | Putamen | PUT |
| 79, 80 | Lenticular nucleus, Pallidum | Pallidum | PAL |
| 81, 82 | Thalamus | Thalamus | THA |
| 83, 84 | Heschl’s gyrus | Heschl | HES |
| 85, 86 | Superior temporal gyrus | Temporal_Sup | STG |
| 87, 88 | Temporal pole: superior temporal gyrus | Temporal_Pole_Sup | TPOsup |
| 89, 90 | Middle temporal gyrus | Temporal_Mid | MTG |
| 91, 92 | Temporal pole: middle temporal gyrus | Temporal_Pole_Mid | TPOmid |
| 93, 94 | Inferior temporal gyrus | Temporal_Inf | ITG |

**Table S2.** A summary of the demographic information and the psychiatric diagnosis in the present study.

| **Sites** | **Group** | **Age (years)** | **Sex (male/female)** | **Education (years)** | **Medication (yes / no)** | **HAMD** | **BDI** | **Duration of illness** | **First episode (yes / no)** | **Mean FD** |
| --- | --- | --- | --- | --- | --- | --- | --- | --- | --- | --- |
| **Taiwan** | Healthy | 49.18±8.58 | 60 / 36 | 15.04 ± 3.83 | / | / | / | / | / | 0.133 ± 0.054 |
|  | Patient | 52.64±14.86 | 33 / 21 | 12.66 ± 3.95 | 54 / 0 | 9.34 ± 6.99 | / | 8.63 ± 6.92 | 0 / 54 | 0.116 ±0.056 |
|  | Statistic  (t / p) | -1.810 / 0.072 | 0.028 / 0.866 | 3.60 / 4.3e-4 |  | / | / | / | / | 1.833 / 0.069 |
| **Xinan** | Healthy | 39.65 ± 15.80 | 166 / 88 | 13.01 ± 3.89 | / | / | / | / | / | 0.133 ±0.063 |
|  | Patient | 38.74 ± 13.65 | 183 / 99 | 11.91 ± 3.58 | 157 / 125 | 20.8 ± 5.87 | 20.4 ± 9.33 | 4.16 ± 5.51 | 209 / 49 | 0.125 ± 0.054 |
|  | Statistic  (t / p) | 0.719 / 0.472 | 0.013 / 0.911 | 3.41 / 6.9e-4 | / | / | / | / | / | 1.729 / 0.084 |

Age, education, HAMD, BDI, duration of illness and Mean FD are presented in mean ± SD.

HAMD = Hamilton Depression Rating Scale;

BDI = Beck Depression Inventory

Mean FD = mean framewise displacements.

**Table S3.** Functional connectivity of the significant posterior cingulate cortex voxels with different brain areas in the controls, in the whole group of patients with depression (i.e. medicated and unmedicated, corresponding to Fig. 1), and the t value and p value of the difference between patients and controls. A positive t value indicates a greater functional connectivity in patients.

| **Regions** | **Posterior cingulate cortex** | | | | |
| --- | --- | --- | --- | --- | --- |
|  | **FC of controls** | **FC of patients** | **t value** | **p value** |  |
| Frontal_Sup_2_L | 0.515 | 0.467 | -4.275 | 1.91E-05 |  |
| Frontal_Mid_2_L | 0.480 | 0.444 | -3.023 | 2.50E-03 |  |
| Frontal_Mid_2_R | 0.239 | 0.306 | 4.049 | 5.14E-05 |  |
| Frontal_Inf_Oper_R | 0.075 | 0.150 | 4.452 | 8.52E-06 |  |
| Frontal_Inf_Tri_R | 0.079 | 0.152 | 4.324 | 1.53E-05 |  |
| Frontal_Inf_Orb_2_R | 0.144 | 0.195 | 3.651 | 2.62E-04 |  |
| Frontal_Sup_Medial_L | 0.613 | 0.584 | -2.189 | 0.029 |  |
| OFClat_R | 0.234 | 0.288 | 3.186 | 1.44E-03 |  |
| Cingulate_Ant_L | 0.428 | 0.357 | -4.216 | 2.49E-05 |  |
| Cingulate_Ant_R | 0.435 | 0.373 | -3.800 | 1.45E-04 |  |
| Cingulate_Post_L | 0.989 | 0.989 | -2.146 | 0.032 |  |
| Cingulate_Post_R | 0.969 | 0.967 | -1.688 | 0.091 |  |
| Fusiform_L | 0.162 | 0.168 | 1.077 | 0.282 |  |
| Fusiform_R | 0.095 | 0.108 | 1.213 | 0.225 |  |
| Precuneus_L | 0.835 | 0.822 | -2.717 | 6.59E-03 |  |
| Temporal_Pole_Mid_L | 0.298 | 0.344 | 3.212 | 1.32E-03 |  |
| Temporal_Inf_L | 0.082 | 0.086 | 0.789 | 0.430 |  |

References

1. Hamilton, M. A rating scale for depression. *J. Neurol. Neurosurg. Psychiatry* **23**, 56-62 (1960).

2. Beck, A.T. & Beamesderfer, A. Assessment of depression: the depression inventory. *Mod. Probl. Pharmacopsychiatry* **7**, 151-169 (1974).

3. Cheng, W.*, et al.* Medial reward and lateral non-reward orbitofrontal cortex circuits change in opposite directions in depression. *Brain* **139**, 3296-3309 (2016).

4. Chao-Gan, Y. & Yu-Feng, Z. DPARSF: a MATLAB toolbox for “pipeline” data analysis of resting-state fMRI. *Front. Syst. Neurosci.* **4**, 13 (2010).

5. Friston, K.J., Williams, S., Howard, R., Frackowiak, R.S. & Turner, R. Movement-related effects in fMRI time-series. *Magn. Reson. Med.* **35**, 346-355 (1996).

6. Power, J.D.*, et al.* Methods to detect, characterize, and remove motion artifact in resting state fMRI. *Neuroimage* **84**, 320-341 (2014).

7. Tomasi, D. & Volkow, N.D. Laterality patterns of brain functional connectivity: gender effects. *Cereb. Cortex* **22**, 1455-1462 (2012).

8. Geerligs, L., Renken, R.J., Saliasi, E., Maurits, N.M. & Lorist, M.M. A Brain-Wide Study of Age-Related Changes in Functional Connectivity. *Cereb. Cortex* **25**, 1987-1999 (2015).

9. Power, J.D., Barnes, K.A., Snyder, A.Z., Schlaggar, B.L. & Petersen, S.E. Spurious but systematic correlations in functional connectivity MRI networks arise from subject motion. *Neuroimage* **59**, 2142-2154 (2012).

10. Rolls, E.T., Joliot, M. & Tzourio-Mazoyer, N. Implementation of a new parcellation of the orbitofrontal cortex in the automated anatomical labeling atlas. *Neuroimage* **122**, 1-5 (2015).
